# First finding of coesite in the eclogitic continental crust of the Sesia Zone, Italian Western Alps

**DOI:** 10.1093/nsr/nwad074

**Published:** 2023-03-20

**Authors:** Giorgio V Dal Piaz

**Affiliations:** Accademia Nazionale dei Lincei, Italy

The Western Alps provide classic examples of eclogite facies subduction metamorphism widely preserved in Adria- and Europe-derived continental nappes and interposed ophiolitic units derived from the suture of the Piedmont-Ligurian cean, all shown in the Structural Model of Italy [1]. The recognition of eclogitic units dates to the end of the 19th century when the Western Alps were accurately surveyed at 1 : 25.000 scale by the Royal Geological Service for the new Geological Map of Italy. This immense work was synthesized in the *Carta Geologica delle Alpi Occidentali*, an amazing map at 1 : 400.000 scale published in 1908, showing in particular the Eclogitic micaschist complex of the Sesia-Val di Lanzo (shortly Sesia) Zone which is presently studied by Chen *et al.* [2]. Previously envisaged as pre-Permian, the metamorphism of this eclogitic continental complex was re-classified by Dal Piaz *et al.* [3] to the Late Cretaceous and referred to the south-eastward subduction of a continental and oceanic lower plate below the Adriatic active margin, clearly before the continental collision, while mineral assemblages and petrological estimates were provided by Compagnoni *et al.* [4]. All this demonstrated that even large fragments of light continental crust had been forcibly dragged to subcrustal depths under the low thermal-regime which is a peculiar mark of lithospheric subduction [3,4]. The first finding of coesite in the Western Alps was due to Chopin [cited in Ref. 2], as evidence of UHP metamorphism in the continental crust of the Penninic (European) southern Dora Maira nappe. Subsequently, coesite and microdiamonds were found by Reinecke [cited in Ref. 2] and Frezzotti *et al.* [cited in Ref. 2] in eclogitic metabasalts of Cignana (Aosta valley), within the Zermatt-Saas ophiolitic unit below the Austroalpine Dent Blanche nappe. Two other coesite sites have been recently discovered by Manzotti *et al.* [5], in the northern Dora-Maira, and by Chen *et al.* [2] in the Eclogitic micaschist complex of the Sesia Zone, Argand's ‘root zone’ of Dent Blanche nappe [3,4]. Among them, Chen's paper [2] deserves to be highlighted for its original contribution to the geochronology, geochemistry and petrology on leucocratic metagranitoids of the Sesia eclogitic complex in the lower Aosta valley: this unit underwent UHP metamorphism at 76.0 ± 1.0 Ma (U-Pb zircon ages), 2.8–3.3 GPa and 450–520°C [2], corroborating previous regional dating and P-T estimates [5, 6 and references therein]. In this view, all the continental and oceanic units of the Austroalpine-Penninic collisional wedge [7] experienced subduction and a similar UHP metamorphism [2,5,6]. Kinematic models are thoroughly discussed by Chen *et al.* [2], favoring a subduction of continental lithosphere dynamically governed by far-field compression [2], similar to tectonic erosion [8] and the forced prograde evolution of previous models [3,6,7]. A similar modern study [2] would be welcome on the eclogitic minor units of the Dent Blanche nappe, as well as on the blueschist facies relics in felsic and mafic basement rocks of the Briançonnais nappe system [1,7,8].
